# Concentrations of PGE_2_ and TXB_2_ in the Eyes of Mice with Disseminated Acanthamoebiasis

**DOI:** 10.3390/pathogens11040438

**Published:** 2022-04-04

**Authors:** Karolina Kot, Daria Kołodziej, Patrycja Kupnicka, Danuta Izabela Kosik-Bogacka, Natalia Łanocha-Arendarczyk

**Affiliations:** 1 Department of Biology and Medical Parasitology, Pomeranian Medical University in Szczecin, Powstańców Wielkopolskich 72, 70-111 Szczecin, Poland; karolina.kot@pum.edu.pl (K.K.); daria_kolodziej@wp.pl (D.K.); natalia.lanocha.arendarczyk@pum.edu.pl (N.Ł.-A.); 2 Department of Biochemistry and Medical Chemistry, Pomeranian Medical University in Szczecin, Powstańców Wielkopolskich 72, 70-111 Szczecin, Poland; pkupnicka@gmail.com; 3 Independent Laboratory of Pharmaceutical Botany, Pomeranian Medical University in Szczecin, Powstańców Wielkopolskich 72, 70-111 Szczecin, Poland

**Keywords:** *Acanthamoeba* spp., disseminated acanthamoebiasis, eyes, prostaglandin E_2_, thromboxane B_2_

## Abstract

Previous studies have shown that *Acanthamoeba* spp. may invade the eyes by migrating along the optic nerve to the eyes from the brain. This study aimed to confirm the presence of inflammation in the eyes of mice with disseminated acanthamoebiasis by examining prostaglandin E_2_ (PGE_2_) and thromboxane B_2_ (TXB_2_) concentrations in the eyes of immunocompetent and immunocompromised mice intranasally inoculated with *Acanthamoeba* spp. The PGE_2_ concentration was statistically significantly lower in the immunocompromised amoebae-infected mice on 8 dpi compared with the noninfected group of animals, and it was higher in the eyes of immunosuppressed amoebae-infected mice on 16 dpi than in the control group of animals. There was a statistically significant lower TXB_2_ concentration in the eyes of immunocompetent infected mice compared with the noninfected group on 8 dpi. However, on 24 dpi, we noted statistically significant higher TXB_2_ levels in the immunocompetent infected mice than in the control group. In immunocompromised mice, there was a lower TXB_2_ level on 8 dpi than in control mice. This study confirmed the existence of an inflammatory process in the eyes of immunocompetent and immunocompromised mice infected with *Acanthamoeba* spp. without damaged corneas.

## 1. Introduction

Particularly dangerous to the health and life of hosts are microorganisms that can have both free-living and parasitic lifestyles. Such species include amoebae from the *Acanthamoeba* genus. These cosmopolitan organisms occur in water, soil, and air as trophozoites and cysts that are resistant to a wide range of chemical and physical agents [1]. *Acanthamoeba* spp. cause *Acanthamoeba* keratitis (AK), granulomatous amebic encephalitis (GAE), and disseminated acanthamoebiasis [2]. To date, more than 3000 cases of AK have been described in the scientific literature [3]. The pathogenesis of AK is well-known [4] in contrast to the mechanisms of *Acanthamoeba* spp. invasion of the eyes due to disseminated acanthamoebiasis. *Acanthamoeba* spp. migrate with the blood to distant organs [2], but to the eyes, they may migrate from the brain along the optic nerve [5,6]. Pathomorphological changes are not extensive; however, previously, there were some differences at the cellular and molecular levels in groups of animals experimentally infected with *Acanthamoeba* spp. compared with control groups. Increased expression of Toll-like receptors (TLR2 and TLR4) [6] and dysregulation of a nonenzymatic antioxidant and antioxidant enzymes [7] in the group of *Acanthamoeba* spp.-infected animals compared with noninfected hosts were reported. Under the condition of increased oxidative stress, the expression and activation of cyclooxygenases (COXs) are altered [8]. Cyclooxygenase isoenzymes (COX-1 and -2) biosynthesize a conversion reaction in which the substrate is arachidonic acid and the products are prostaglandins (PGs). COXs play an important role in eye health and disease. The prostaglandins are composed of five different classes, PGD_2_, PGE_2_, PGF_2_, PGI_2_, and thromboxane A (TXA_2_) [8,9]. Prostaglandin E_2_ is a metabolite of COX-2. However, in the eye, PGE_2_ formation may be attributed to both COX-1 and COX-2 [10]. PGE_2_ is one of the most studied PGs. It is responsible for the generation of fever as well as pain and neurotransmitter modulation. PGE_2_ has been studied for its role in the eye, particularly in the lowering of intraocular pressure (IOP) [11]. Thromboxane A_2_ (TXA_2_) is the prostanoid product of COX-1 [12]. TXA_2_ contains an unstable ether linkage that is rapidly hydrolyzed to form the biologically inert TXB_2_. Increased levels of TX are observed in inflammatory diseases. TXB_2_ may lower IOP. Moreover, TXA_2_ does not have a direct role in glaucoma, but the physical interaction of TXA_2_ with a gene product implicated in open-angle glaucoma pathology was previously shown [13].

PGs play a role in parasitic diseases, such as amoebiasis caused by *Entamoeba histolytica*, leishmaniasis, Chagas disease, malaria, and pulmonary acanthamoebiasis [14,15,16,17,18]. The aim of this study was to confirm the existence of inflammation in the eyes of mice with disseminated acanthamoebiasis. The purpose of this study was achieved by the examination of concentrations of cyclooxygenase products, prostaglandin E_2_ (PGE_2_) and thromboxane B_2_ (TXB_2_), in the eyes of immunocompetent and immunocompromised mice intranasally inoculated with *Acanthamoeba* spp. To date, no studies have addressed the implications of the role of COXs’ metabolites in *Acanthamoeba* spp. eye infections.

## 2. Results

### 2.1. Concentration of PGE_2_

**Figure 1:**
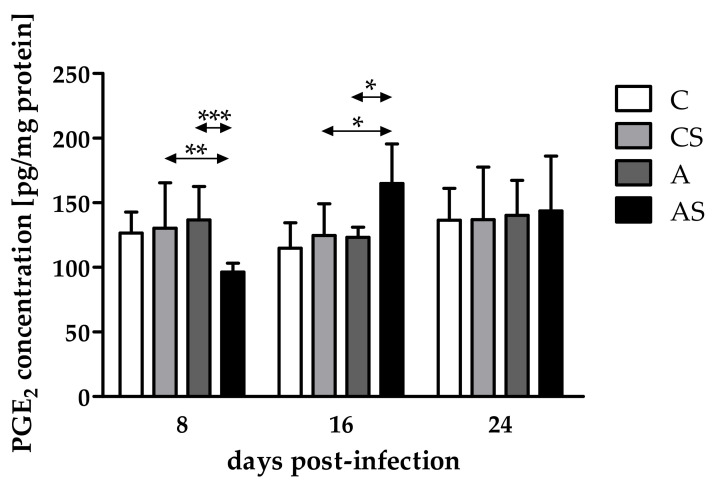
Concentrations of prostaglandin E_2_ (PGE_2_) in the eyes of mice at 8, 16, 24 days post *Acanthamoeba* spp. infection (dpi) related to immunological status of the host. Data represent mean ± standard deviation for six independent experiments (C, immunocompetent control, noninfected mice; CS, immunosuppressed control, noninfected mice; A, immunocompetent mice infected with *Acanthamoeba* spp.; AS, immunosuppressed mice infected with *Acanthamoeba* spp.; * statistical difference at *p* < 0.05; ** statistical difference at *p* < 0.01; *** statistical difference at *p* < 0.001).

Comparing PGE_2_ concentrations in the eyes of immunocompromised *Acanthamoeba* spp.-infected mice on different days after infection, differences occurred and were statistically significantly different (H = 14.10, *p* < 0.001). There was a decrease in the PGE_2_ concentration at 8 dpi, an increase at 16 dpi, and a re-decrease at 24 dpi. At 8 dpi, the PGE_2_ concentration was statistically significantly lower in the immunocompromised parasite-infected mice than in the control group of animals (U = 10, *p* < 0.01; Figure 1). However, at 16 dpi, a higher PGE_2_ level was observed in the eyes of immunosuppressed infected hosts than in noninfected animals (U = 23, *p* = 0.01; Figure 1).

Taking into account host immune status, the PGE_2_ concentration was lower in the eyes of immunosuppressed infected mice than in immunocompetent infected mice at 8 dpi (U = 2, *p* < 0,001; Figure 1), and it was higher in the eyes of immunocompromised mice than in immunocompetent hosts at 16 dpi (U = 4, *p* = 0.02; Figure 1). At 24 dpi, the concentration of PGE_2_ was at a similar level in the eyes of immunosuppressed and immunocompetent infected hosts.

### 2.2. Concentration of TXB_2_

**Figure 2:**
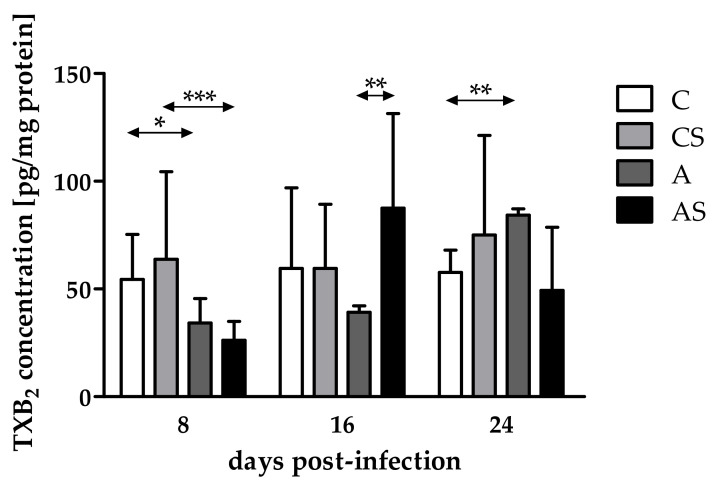
Concentrations of thromboxane B_2_ (TXB_2_) in the eyes of mice at 8, 16, 24 days post *Acanthamoeba* spp. infection (dpi) related to immunological status of the host. Data represent mean ± standard deviation for six independent experiments (C, immunocompetent control, noninfected mice; CS, immunosuppressed control, noninfected mice; A, immunocompetent mice infected with *Acanthamoeba* spp.; AS, immunosuppressed mice infected with *Acanthamoeba* spp.; * statistical difference at *p* < 0.05; ** statistical difference at *p* < 0.01; *** statistical difference at *p* < 0.001).

The concentration of TXB_2_ in the eyes of immunocompetent *Acanthamoeba* spp.-infected mice decreased at 8 dpi and increased at 16 and 24 dpi (H = 12.16, *p* < 0.01). We noted a statistically significant lower TXB_2_ concentration in the eyes of immunocompetent infected mice than in the noninfected group at 8 dpi (U = 13, *p* = 0.02; Figure 2). Additionally, we found statistically significant higher TXB_2_ levels in the immunocompetent infected mice than in the control group at 24 dpi (U = 0, *p* < 0.001; Figure 2).

Comparing TXB_2_ concentrations in the eyes of immunocompromised mice on different days after infection, differences occurred and were statistically significant (H = 12.38, *p* < 0.01). The concentration of TXB_2_ decreased at 8 dpi, increased at 16 dpi, and re-decreased at 24 dpi. Moreover, we found a statistically significant lower TXB_2_ level in the immunosuppressed infected mice than in the uninfected group at 8 dpi (U = 9, *p* < 0.001; Figure 2).

Taking into account host immune status, TXB_2_ concentrations were at the same level in the eyes of immunosuppressed and immunocompetent infected mice at 8 dpi. At 16 dpi, TXB_2_ levels increased in the immunosuppressed mice compared with the immunocompetent animals, while at 24 dpi, TXB_2_ levels decreased in the immunosuppressed hosts. The difference was statistically significant only at 16 dpi (U = 0, *p* < 0.01; Figure 2). 

## 3. Discussion

In our previous studies, we demonstrated that *Acanthamoeba* spp. can invade the eyes of a host without a damaged cornea. Amoebae may migrate from the brain to the eyes along the optic nerve in hosts with disseminated acanthamoebiasis [6]. In the scientific literature, intraocular colonization secondary to disseminated acanthamoebiasis was reported only once in humans in *post mortem* examinations [5], but the reproduction of this finding in an animal model [6] warns that the situation could occur with more frequency. Moreover, among symptoms occurring during GAE, eye distention and photophobia with blurred vision are sometimes described [19,20,21]. We speculate that in these patients with GAE and vision problems, *Acanthamoeba* spp. invaded the eyes. However, we know neither what circumstances have to occur to make amoebae migrate through the optic nerve nor how fast they invade the eyes. In live patients, we found no case reports concerning eye invasions by amoebae in GAE or disseminated acanthamoebiasis. Most probably, it is associated with high mortality in patients with GAE, and that diagnostic is often confirmed *post mortem* [22]. 

Symptoms occurring with *Acanthamoeba* spp. infection are associated with an ongoing inflammatory process. The proteins involved in this process are PGs, products of the reaction carried out by cyclooxygenases [23,24]. Under normal physiological conditions, PGs have crucial homeostatic functions, including maintaining IOP. However, prostaglandins are also involved in some pathological conditions, including ocular inflammation [25]. PGs increase vasodilation, facilitate leukocyte migration, and damage the blood–ocular barrier [26]. Thromboxanes are important mediators of inflammation. However, data concerning the role of TXB_2_ in the eyes and the influence of TXB_2_ on the pathogenesis of various eye diseases is limited. TXB_2_ may be involved in inflammation in glaucoma and dry eye [13,27]. The upregulation of TXB_2_ was demonstrated in response to a cornea infection with *Pseudomonas aeruginosa* [28]. Thromboxane may be involved in host–parasite interactions. In the eyes of mice with disseminated acanthamoebiasis, changes in TXB_2_ levels were observed in immunocompetent hosts on 8 and 24 dpi and in immunocompromised mice at the beginning of infection. 

PGE_2_ is important in the inflammatory process [29]. There is experimental evidence indicating the involvement of PGE_2_ in the pathogenesis of various eye diseases, including diabetic retinopathy and glaucoma [11,23]. Through studying diabetic retinopathy in animal models, it has been reported that retinal cells constantly increase COXs and PGE_2_ enzyme levels [30,31]. Schoenberger et al. [23] found higher PGE_2_ levels in diabetic human eyes. Lekhanont et al. [32] reported a positive correlation between the PGE_2_ concentration in tears and the symptoms of dry eye. Considering parasitic diseases, it was suggested that PGE_2_ is involved in the pathogenesis of ocular toxoplasmosis [33]. PGE_2_ may modulate a host’s immune system in terms of the inhibition of nitrogen oxide (NO) production, immunosuppression, and the inhibition of interferon (IFN) and apoptotic pathways. Additionally, PGE_2_ may play a role in infections by the induction of autoimmune disorder and disruption of signaling via TLRs [34]. In this study, we also noted the role of PGE_2_ in the pathomechanism and pathophysiology of *Acanthamoeba* spp. invasion in the eyes of immunosuppressed mice. Degraaf et al. [35] reported that PGE_2_ reduces TLR4 expression, which agrees with our research. In immunosuppressed mice, an increased level of TLR4 expression was not observed [6].

The increased production of PGE_2_ in pathological conditions is mostly regulated by the induction of the *COX-2* gene [36]. In human keratinocytes, increased intracellular ROS as a result of mechanical injury stimulates PGE_2_ production via the activation of COX-2 [37]. In pulmonary acanthamoebiasis, differences in COX-1 and COX-2 expressions were only observed between immunocompetent infected and noninfected mice. Interestingly, there were no differences in PGE_2_ and TXB_2_ concentrations between amoebae-infected and noninfected mice regardless of the immunological status of the animals [18]. Despite antioxidant dysregulation in both immunocompetent and immunosuppressed eyes of mice infected with *Acanthamoeba* spp. [7], in our study, we noted differences in TXB_2_ in immunocompetent and immunosuppressed mice, while differences in PGE_2_ concentration were observed only in immunosuppressed mice. We suggest that in the immunocompetent mice, there was a blockage of PGE_2_ production. Strong et al. [38] reported that suppressing PGE_2_ production leads to a decrease in proinflammatory cytokines levels and provides a survival advantage to the host.

PGE_2_ is expressed in the human cornea, iris, trabecular meshwork, ciliary body, conjunctiva, and retina [11]. The retinal pigment epithelium is capable of inducing the differentiation of T cells into regulatory T cells through the expression of prostaglandin E_2_. The RPE induces the production of PGE_2_, thereby increasing granulocyte maturation and the inflammatory process [38]. Wang et al. [39] found retinal structural abnormalities, retinal edema, and neovascularization in the eyes of rats with diabetic retinopathy after PGE_2_ treatment compared with rats with diabetic retinopathy not treated with PGE_2_. In our study, we found a higher level of PGE_2_ in immunosuppressed mice infected with the parasite on 16 dpi compared with the noninfected group. In this infected group of mice, we also found an increased thickness in the outer nuclear layer of the retina, vacuolization inside the outer plexiform layer, and invagination of the Bowman’s membrane into the substantia propria. However, an increased thickness in the layers of the retina was observed in immunocompetent infected mice at 16 and 24 dpi as well as in immunosuppressed infected mice at 24 dpi [7], in which increased PGE_2_ levels were not found. 

Yamanishi et al. [24] found decreased production of PGE_2_ in the tears of patients with severe conjunctivitis after dexamethasone administration. Fuller et al. [40] reported that dexamethasone inhibits the production of thromboxane B_2_. Dexamethasone and methylprednisolone are immunosuppressive drugs known as corticosteroids. In our study, we observed decreased PGE_2_ levels in the immunocompromised infected mice compared with the immunocompetent infected hosts on 8 dpi. On 16 dpi, PGE_2_ and TXB_2_ levels were higher in the AS group than in the A group. 

Ocular pathology in disseminated acanthamoebiasis may be affected by a Th17-dependent immune response. It was suggested that *Toxoplasma gondii* induce an IL-17 increase, which exacerbates pathology and inflammation [41]. This cytokine may allow antigens, antibodies, and activated immune cells to cross the blood–retinal barrier, leading to increased inflammation and subsequent tissue damage [33,42]. In sharp contrast, Suryawanshi et al. [43] reported that IL-17 plays a significant role in host protection against *Acanthamoeba* spp. invasion. Łanocha-Arendarczyk et al. [44] showed that in disseminated acanthamoebiasis in immunocompetent hosts, parasites induce Th1, Th2, and Th17 responses. Meanwhile, in immunocompromised hosts, *Acanthamoeba* spp. induces strong immunity mediated by Th1 cells without Th17 involvement. IL-17 activates the TXB_2_ pathway [45], while prostaglandin E_2_ is essential for the production of IL-17, the Th17 effector cytokine [46]. However, Dejani et al. [47] reported that Th17 cell differentiation during intestinal *Citrobacter rodentium* infection is inhibited by PGE_2_. In our study, we did not examine IL-17 levels in the eyes of mice infected with amoebae, but it should be done in a future study to confirm our suggestions.

## 4. Materials and Methods

### 4.1. Animal Model

Approval was obtained from the ethics committees in Szczecin (Resolution No. 29/2015, 22 June 2015) and Poznań (Resolution No. 64/2016, 9 September 2016) for the study. The *Acanthamoeba* strain was classified as a T16 genotype, and it was isolated from a patient who suffered from acute myeloid leukemia (AML). 

Ninety-six male mice of strain BALB/c, aged 6–10 weeks, with an average weight of 23 g, were used for the study. The animals were obtained from the Center of Experimental Medicine, Medical University of Bialystok. They were properly examined by a veterinarian who issued certificates of their health status. 

**Figure 3:**
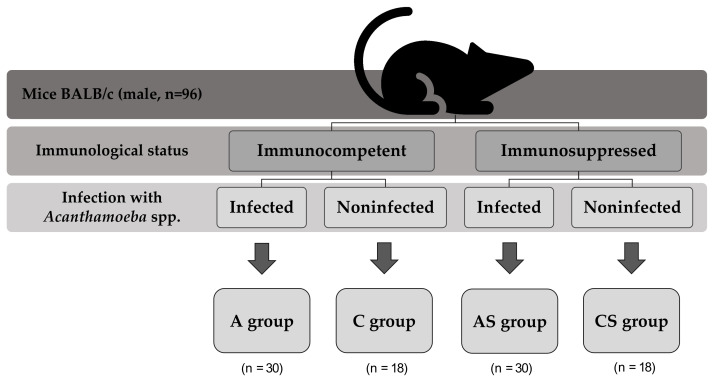
Division of mice in the experiment.

Animals were divided into four groups on the basis of their immune status and parasite infection (Figure 3). 

For four days prior to inoculation of mice with *Acanthamoeba* spp., AS and CS animals were administered 0.22 mg (10 mg/kg body weight, b.w.) of methylprednisolone dissolved in 0.1 mL of 0.1% saline to induce immunosuppression. Infections of A and AS groups were performed by intranasal inoculation with 3 µL of a suspension containing 10–20,000 amoebae in accordance with the animal model used by Górnik and Kuźna-Grygiel [48]. C and CS groups received 3 µL of saline (0.9% NaCl).

Animals were monitored daily for clinical signs of *Acanthamoeba* spp. infection. We observed higher activity, changes in fur aspect, and ataxia in some mice. All observations of each mouse in each group were presented in previous papers [18,49]. 

Animals were injected intraperitoneally (i.p.) with sodium pentobarbital (2 mL/kg b.w.) and were sacrificed on days 8, 16, and 24 post *Acanthamoeba* spp. infection (dpi). Eyes were collected from mice using sterilized equipment. Confirmation of *Acanthamoeba* spp. infection was determined by amoeba re-isolation from the organs, which consisted of placing the eyes and optic nerve on a plate with non-nutrient agar and bacteria and then incubating the plate for 10 days at 41 °C [6]. Eyes for biochemical analysis were fixed in liquid nitrogen and stored at −80 °C.

### 4.2. Homogenization

Frozen eyes were placed individually in a metal homogenizer cooled in liquid nitrogen and then fragmented thoroughly by hitting the metal mandrel, which was also cooled in liquid nitrogen, several times with a hammer. The crushed and frozen eye fragments were transferred with a liquid-nitrogen-cooled spoon into Eppendorf test tubes. The homogenizer and hammer were thoroughly cleaned with ethanol after each use. The prepared samples were stored at −80 °C.

### 4.3. Total Protein Concentration

To each sample, 200 µL of buffer (150 mM NaCl, 50 mM Tris-HCl, and 0.5% Triton X-100) was added. Then, they were vortexed and incubated in an ice cartridge for 15 min. The samples were then centrifuged (14,000 rpm for 20 min at 4 °C), and the supernatant was gently extracted. Total protein concentration in the supernatant was examined by the bicinchoninic acid method using a commercial test (MicroBCA Protein Assay Kit, Thermo Scientific, Pierce Biotechnology, Waltham, MA, USA). The absorbance of the samples was then measured spectrophotometrically using a Biochrom EZ Read 2000 plate reader at 562 nm. The essence of this method is the reduction reaction of Cu2+ to Cu+ ions in an alkaline medium and the formation of a colored BCA complex with Cu+ ions, which shows an absorbance maximum at 562 nm. The absorbance value is directly proportional to the total protein concentration.

### 4.4. PGE_2_ and TXB_2_ Concentrations

PGE_2_ and TXB_2_ were extracted from eye sample homogenates using Bakerbond columns (Witko Group, Łódź, Poland). The concentration of PGE_2_ was measured using the Prostaglandin E_2_ EIA Kit according to the manufacturer’s procedure (Cayman, Ann Arbor, MI, USA). TXB_2_ concentration was measured using the Thromboxane B_2_ EIA Kit assay according to the manufacturer’s procedure (Cayman, Ann Arbor, MI, USA). Results were read using a microplate reader (EZ Read 2000, Biochrom Ltd., Cambridge, UK) at 420 nm. The concentrations of PGE_2_ and TXB_2_ were expressed in pg/mg protein.

### 4.5. Statistical Analysis

Statistical analysis was performed using Microsoft Excel 2016, StatSoft Statistica 12.0, and GraphPad 5.0. We calculated the arithmetic mean (AM) and standard deviation of the arithmetic mean (SD). Because the data did not follow a normal distribution, nonparametric tests were used for statistical analysis. The Mann–Whitney U test (U) was used to compare differences between two study groups (*Acanthamoeba* spp. infection and immune status), and the Kruskal–Wallis test (H) was used to compare differences among three study groups (days after infection). Significant statistical differences were assumed when *p* < 0.05.

## 5. Conclusions

The pathomechanism and pathophysiology of disseminated acanthamoebiasis accompanied by an infection of amoebae to the eyes, is still not fully understood. In the present study, we confirmed the inflammatory process in the eyes of immunocompetent and immunocompromised mice infected with *Acanthamoeba* spp. without damaged cornea. In immunocompetent infected mice, the main mediator of inflammation is TXB2, while in immunosuppressed infected mice, both TXB2 and PGE2 are mediators of inflammation.

## Data Availability

Derived data supporting the findings of this study are available from the corresponding author (D.K.-B.) upon request.
